# NEXUS trial: a multicenter phase II clinical study evaluating the efficacy and safety of the perioperative use of encorafenib, binimetinib, and cetuximab in patients with previously untreated surgically resectable *BRAF* V600E mutant colorectal oligometastases

**DOI:** 10.1186/s12885-023-11311-5

**Published:** 2023-08-21

**Authors:** Shin Kobayashi, Hideaki Bando, Akinobu Taketomi, Takeshi Takamoto, Eiji Shinozaki, Manabu Shiozawa, Hiroki Hara, Kentaro Yamazaki, Koji Komori, Nobuhisa Matsuhashi, Takeshi Kato, Yoshinori Kagawa, Mitsuru Yokota, Eiji Oki, Keigo Komine, Shinichiro Takahashi, Masashi Wakabayashi, Takayuki Yoshino

**Affiliations:** 1 Department of Hepatobiliary and Pancreatic Surgery, National Cancer Center Hospital East, 6-5-1 Kashiwanoha, Kashiwa, Chiba 2770882 Japan; 2 Department of Gastroenterology and Gastrointestinal Oncology, National Cancer Center Hospital East, Kashiwa, Japan; 3 Department of Gastroenterological Surgery I, Hokkaido University Hospital, Sapporo, Japan; 4 Department of Hepatobiliary and Pancreatic Surgery, National Cancer Center Hospital, Tokyo, Japan; 5 Gastrointestinal Oncology Department, The Cancer Institute Hospital of JFCR, Tokyo, Japan; 6 Department of Gastrointestinal Surgery, Kanagawa Cancer Center, Yokohama, Japan; 7 Gastroenterological Department, Saitama Cancer Center, Ina, Japan; 8 Division of Gastrointestinal Oncology, Shizuoka Cancer Center, Shizuoka, Japan; 9 Department of Gastroenterological Surgery Aichi Cancer Center Hospital, Nagoya, Japan; 10 Department of Gastroenterological Surgery, Pediatric Surgery, Gifu University Hospital, Gifu, Japan; 11 Department of Surgery, National Hospital Organization Osaka National Hospital, Osaka, Japan; 12 Department of Gastroenterological Surgery, Osaka General Medical Center, Osaka, Japan; 13 Department of General Surgery, Kurashiki Central Hospital, Kurashiki, Japan; 14 Department of Surgery and Science, Graduate School of Medical Sciences, Kyushu University, Fukuoka, Japan; 15 Department of Clinical Oncology, Tohoku University Hospital, Sendai, Japan; 16 Department of Gastroenterological Surgery, Tokai University School of Medicine, Isehara, Japan; 17 Division for the Promotion of Drug and Diagnostic Development, National Cancer Center Hospital East, Kashiwa, Japan

**Keywords:** BRAF V600E, Colorectal cancer, Oligometastases, Resectable, Encorafenib, Binimetinib, Cetuximab, BEACON

## Abstract

**Background:**

The optimal treatment strategy for resectable *BRAF* V600E mutant colorectal oligometastases (CRM) has not been established due to the rarity and rapid progression of the disease. Since the unresectable recurrence rate is high, development of novel perioperative therapies are warranted. On December 2020, the BEACON CRC triplet regimen of encorafenib, binimetinib, and cetuximab was approved for unresectable metastatic colorectal cancer in Japan.

**Methods:**

The NEXUS trial is a multicenter phase II clinical study evaluating the efficacy and safety of the perioperative use of encorafenib, binimetinib, and cetuximab in patients with previously untreated surgically resectable *BRAF* V600E mutant CRM. The key inclusion criteria are as follows: histologically diagnosed with colorectal adeno/adenosquamous carcinoma; *RAS* wild-type and *BRAF* V600E mutation by tissue or blood; and previously untreated resectable distant metastases. The triplet regimen (encorafenib: 300 mg daily; binimetinib: 45 mg twice daily; cetuximab: 400 mg/m^2^, then 250 mg/m^2^ weekly, 28 days/cycle) is administered for 3 cycles each before and after curative resection. The primary endpoint of the study is the 1-year progression-free survival (PFS) rate and the secondary end points are the PFS, disease-free survival, overall survival, and objective response rate. The sample size is 32 patients. Endpoints in the NEXUS trial as well as integrated analysis with the nationwide registry data will be considered for seeking regulatory approval for the perioperative use of the triplet regimen.

**Discussion:**

The use of the triplet regimen in the perioperative period is expected to be safe and effective in patients with resectable *BRAF* V600E mutant CRM.

**Trial registration:**

jRCT2031220025, April. 16, 2022.

## Backgrounds

BRAF is a serine/threonine kinase that belongs to the *RAF* family that was first reported in the early 2000s [1, 2]. It is downstream of *RAS* in the MAPK signaling pathway, which is involved in the regulation of cellular functions, including cell proliferation. Among the *BRAF* mutations, V600E mutation can enhance BRAF kinase activity up to 700-fold of that of the wild-type. *BRAF* V600E mutant metastatic colorectal cancer (mCRC) accounts for 5%–10% of all cases of mCRC and exhibits distinct clinicopathologic features, including a propensity for the right side of the colon, poor histological differentiation, peritoneal dissemination, and high microsatellite instability [3]. Prognosis of unresectable *BRAF* V600E mutant mCRC is poor compared with that of *RAS*-mutant and *RAS*/*BRAF* wild-type; the median overall survival (OS) is 9.2–14.1 months [4–7].

Recently, the BEACON CRC trial, which enrolled patients with previously treated unresectable *BRAF* V600E mutant mCRC, demonstrated that the triplet regimen of encorafenib (a *BRAF* inhibitor), binimetinib (an *MEK* inhibitor), and cetuximab (anti-EGFR antibody) as well as the doublet regimen of encorafenib and cetuximab significantly improved both progression-free survival (PFS) and OS compared with standard chemotherapy (FOLFIRI/irinotecan plus cetuximab) [8, 9]. While the hazard ratios (HR) of the triplet regimen and control groups for PFS and OS were 0.42 (95% CI: 0.33–0.53) and 0.60 (95% CI: 0.47–0.75) (*P* < 0.001), respectively, those of the doublet regimen and control groups were 0.44 (95% CI: 0.35–0.55) and 0.61 (95% CI: 0.48–0.77). As the triplet regimen did not exhibit significant survival benefits over the doublet regimen (HR: 0.95, 95% CI: 0.74 – 1.21), the doublet combination has become the standard second-line treatment in the United States and European Union. However, the confirmed objective response rate (ORR) was 26.8% for the triplet regimen and 19.5% for the doublet regimen, and the triplet regimen was potentially more effective than the doublet regimen in patients with remaining primary lesions, ≥ 3 organs involved, and high C-reactive protein levels, i.e., those with a greater tumor load. Moreover, the triplet regimen achieved a deeper response than the doublet regimen (*p* = 0.033). Therefore, both the triplet and doublet regimens have been approved for use in Japan.

The BEACON CRC trial was followed by the ANCHOR CRC trial wherein the effectiveness of the triplet regimen was evaluated in patients with previously untreated unresectable *BRAF* V600E mutant mCRC. The ORR of the triplet regimen was 47.8% in the ANCHOR CRC trial, which was better than that in the BEACON CRC trial [10]. The efficacy and safety of the doublet regimen are being evaluated in the randomized phase III BREAKWATER trial (NCT04607421), which aims to compare the efficacy of doublet regimen with or without chemotherapy (FOLFOX) with the standard chemotherapy alone (FOLFOX/FOLFOXIRI/CAPOX with or without bevacizumab), in patients with previously untreated unresectable *BRAF* V600E mutant mCRC [11, 12].

The optimal treatment strategy for resectable *BRAF* V600E mutant mCRC has not been established due to the rarity (approximately 3%) and rapid progression of the disease [13–16]. Margonis et al. reported that the median disease-free survival (DFS) and OS after hepatectomy for *BRAF* V600E mutant colorectal liver metastases were 9.9 and 26 months, respectively [14]. We also reported that 93.9% of patients who underwent hepatectomy for *BRAF* V600E mutant colorectal liver metastases developed recurrence after a median 5.3 months, and their median OS was 31.1 months [16]. Since the rate of early onset unresectable systemic recurrence was high, we advocated the necessity of developing novel perioperative therapies [17, 18]. Considering that more than 60% of resectable *BRAF* V600E mutant colorectal liver metastases are metachronous within 1 year after the resection of primary CRC for which patients have mostly received adjuvant oxaliplatin-based chemotherapy [16], the BEACON regimen is considered the optimal regimen of choice for perioperative use. Additionally, the BEACON triplet regimen may even be more preferable to the doublet regimen because 40% of resectable *BRAF* V600E mutant mCRC is synchronous with the primary CRC and often involves three organs (i.e., liver, lung, and primary CRC) [16], hence the preference for the triplet regimen [9]. Moreover, the deeper response that was offered by the triplet regimen justifies its perioperative use, especially in the neoadjuvant setting.

**Fig. 1:**
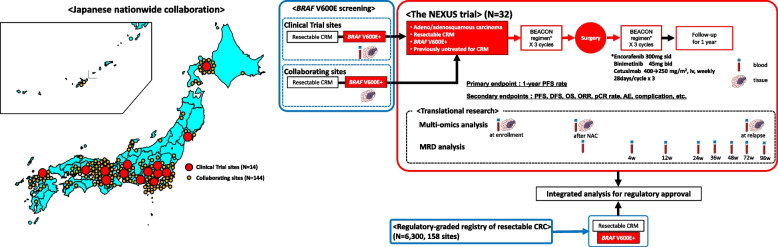
Schematic image of the NEXUS study

Accordingly, we planned a multicenter phase II clinical study to evaluate the efficacy and safety of the perioperative use of encorafenib, binimetinib, and cetuximab in patients with previously untreated but surgically resectable *BRAF* V600E mutant colorectal oligometastases (CRM) (NEXUS trial, jRCT2031220025) (Fig. 1) [19]. Because surgically resectable *BRAF* V600E mutant mCRC is rarer than the unresectable subtype, a randomized trial for this disease is difficult to conduct. In a phase II TRIUMPH study (UMIN000027887), the efficacy and safety of pertuzumab plus trastuzumab were evaluated in patients with previously treated unresectable HER2-positive mCRC [20]. For regulatory approval, we utilized real-world data (RWD) in the regulatory-graded registry (SCRUM-Japan Registry) as an external control [21, 22]. Similarly, a regulatory-graded nationwide clinico-patho-genomic registry of patients scheduled for surgery for resectable CRC was recently established in Japan (GALAXY trial, UMIN000039205) [23, 24]. Therefore, we planned to compare the efficacy and safety of the perioperative use of the triplet regimen with those of the RWD extracted from the registry as an external control.

## Methods/Design

### Patient screening

Patients with resectable CRM at institutions participating in the NEXUS trial were screened for *BRAF* V600E by screening their tissue or blood sample using comprehensive genotyping assay using Guardant360 (Guardant Health, Inc., Redwood City, CA, USA) to detect circulating tumor DNA (ctDNA) (PRECISION study, UMIN000042490) [25]. Aside from the institutions participating in the NEXUS trial, more than 140 institutions all over Japan that participated in the GALAXY trial were encouraged to refer eligible patients with *BRAF* V600E mutant CRM to institutions participating in the NEXUS trial.

### Study design and treatment

The NEXUS trial is a multicenter phase II clinical study that will evaluate the efficacy and safety of the perioperative use of encorafenib, binimetinib, and cetuximab (the BEACON triplet regimen) in patients with previously untreated but surgically resectable *BRAF* V600E mutant CRM. Table 1 shows the eligibility criteria. The key inclusion criteria are as follows: age ≥ 20 years; Eastern Cooperative Oncology Group Performance Status 0 or 1; histologically diagnosed with primary adenocarcinoma or adenosquamous carcinoma of the colon or rectum; *RAS (KRAS/NRAS)* wild-type and *BRAF* V600E mutation upon analysis of tumor tissues or blood specimens; and previously untreated distant metastasis for which macroscopic complete resection (R0/1 resection according to the Japanese guidelines for the treatment of CRC [26]) is possible. Patients in whom R0/1 resection is impossible unless tumor shrinkage or elimination is achieved by chemotherapy were excluded. The triplet regimen (encorafenib: 300 mg daily, binimetinib: 45 mg twice daily, cetuximab: 400 mg/m^2^ of body-surface area as an initial dose, then 250 mg/m^2^ weekly, 28 days in a cycle), [8] as described in the BEACON CRC trial, is offered for 3 cycles each before and after curative resection.

**Table 1:** Eligibility criteria of the NEXUS study

Inclusion criteria
1. Patients who have voluntarily provided written consent for participation
2. Patients aged ≥ 20 years at the time of providing informed consent
3. Patients with ECOG Performance Status of 0 or 1
4. Patients histologically diagnosed as having adenocarcinoma or adenosquamous carcinoma of the colon or rectum as the primary site
5. Patients with *RAS (KRAS/NRAS)* wild-type and *BRAF* V600E mutation as confirmed by analysis of tumor tissues or blood specimens
6. Patients in whom distant metastasis detected by imaging examination within 28 days of the date of enrollment and in whom macroscopic complete resection (R0/1 resection) is possible for all lesions
7. In cases of metachronous distant metastasis, R0/1 resection of the primary tumor has been achieved
8. Patients can tolerate surgery
9. Patients with previously untreated distant metastasis
10. Adequate laboratory test results performed within 28 days before enrollment
11. Patients who can take oral medication
12. Patients who agree to use highly effective contraception
Exclusion criteria
1. Patients with a history of previous treatment with anti-EGFR antibody drugs, RAF inhibitors, or MEK inhibitors
2. Patients with any other unresectable advanced and recurrent cancer
3. Patients in whom R0/1 resection is impossible unless tumor shrinkage or elimination is achieved by chemotherapy
4. Patients with a history or finding of cardiovascular risk
5. Patients with poorly controlled diabetes or other diseases that may interfere with the toxicity evaluation
6. Patients with a history or finding of retinal and neuromuscular diseases
7. Pregnant or breastfeeding women
8. Patients with significant and unstable psychiatric disorders or other medical illnesses
9. Patients who do not intend to adhere to the procedures specified in the protocol
10. Patients with other serious medical illness
11. Patients whose enrollment is deemed inappropriate by the investigators

### Endpoints

The primary endpoint is the 1-year PFS rate, and the secondary endpoints are the PFS, DFS, OS, 1-year PFS rate by central image review, ORR, pathological complete response (CR) rate of distant metastatic lesions and the primary lesion as assessed by pathologists at each institution, protocol treatment completion rate, R0 resection rate, incidence of adverse events, and incidence of surgery-related complications. An exploratory endpoint is the analysis of biomarkers related to the efficacy and toxicity of the protocol treatment. Progression is defined as either progressive disease based on diagnostic imaging in the assessment of overall response according to the revised guidelines on the Response Evaluation Criteria in Solid Tumors version 1.1 [27] during the neoadjuvant treatment, macroscopically incomplete resection (R2 resection), recurrence after surgical resection, or death. The 1-year PFS rate is defined as the proportion of patients who developed progression at the 1-year time point as measured from the date of registration.

### Sample size and statistical analysis

As the 1-year DFS in patients with *BRAF* V600E mutant colorectal liver metastases is 24% in Japan, and the HR of triplet therapy for PFS in the final report of the BEACON CRC trial is 0.42 [9, 16], 25% and 50% were set as the threshold and expected values, respectively, for the 1-year PFS rate in this study. Planned patient accrual was set at 32 patients, with the one-sided significance level at 2.5% and statistical power at 80%.

### Translational analysis

Multi-omics analysis of both tumor tissues and blood specimens at enrollment, resection, and relapse will be performed to investigate the prognostic factors (MONSTAR-2 study, UMIN000043899) [28]. ctDNA is analyzed before surgery and at 4, 12, 24, 36, 48, 72, and 96 weeks postoperatively to evaluate the value of molecular residual disease using a personalized tumor-informed ctDNA assay, Signatera (Natera, Inc., San Carlos, CA, USA) in this disease setting [23].

### Planned regulatory approval

Since the NEXUS trial is a single-arm study, the efficacy and safety data will be compared with those of the RWD in the registry, wherein treatments other than this protocol treatment were administered to the same subjects. PFS, DFS, OS, and incidence of surgery-related complications in the NEXUS trial will be compared with those extracted from the regulatory-graded prospective large-scale nationwide clinico-patho-genomic registry (GALAXY trial) [23, 24]. Endpoints in the NEXUS trial as well as integrated analysis with the registry data will be considered for regulatory approval to expand the indications of the BEACON triplet regimen, including its perioperative use.

## Discussion

Since the discovery of the notorious *BRAF* V600E mutation, many attempts have been made to improve the prognosis of patient with CRC with the said mutation. As its prognosis is, by far, worse in patients with unresectable metastatic disease than in those with locally limited resectable ones, clinical trials focusing on *BRAF* V600E CRC have been conducted for unresectable metastatic disease. However, because most cases of metastatic disease are found with unresectable tumors, no prospective clinical trials have been conducted for metastatic and resectable cases. To the best of our knowledge, the NEXUS trial is the first and the only trial to investigate the efficacy of perioperative targeted therapy for resectable *BRAF* V600E mutant CRM.

While survival outcomes of patients with surgically resected *BRAF* V600E mutant CRM are better than those in medically treated patients [29, 30], upfront resection of resectable *BRAF* V600E mutant CRM also has a poor prognosis [16, 18]. Patients with *BRAF* V600E mutant liver metastases developed early systemic and unresectable recurrences within 8 months after surgery, and the OS was almost identical to that after systemic chemotherapy for unresectable cases. Moreover, since *BRAF* V600E mutant mCRC is a rapidly progressive disease and that causes rapid deterioration of performance status with early relapse after surgery, 20% of patients who underwent upfront hepatectomy could not receive chemotherapy after recurrence. Therefore, the use of neoadjuvant chemotherapy is warranted in this patient cohort [17, 31]. Perioperative chemotherapy might be effective to control micro-metastatic lesions. Amaria et al. conducted a randomized phase II study comparing perioperative doublet therapy with dabrafenib (a *BRAF* inhibitor) and trametinib (an *MEK* inhibitor) and surgery followed by adjuvant therapy in patients with stage III or resectable stage IV malignant melanoma with *BRAF* V600E/K mutation [32]. The study was terminated when statistical evidence of the efficacy was demonstrated in only 21 patients, with the DFS being 19.7 months and 2.9 months for the perioperative doublet therapy and surgery-first groups, respectively (HR: 0.016; 95% CI: 0.00012–0.14). Therefore, the efficacy and safety of the perioperative use of the BEACON regimen are expected in patients with resectable *BRAF* V600E mutant CRM.

Although two decades have passed since the detrimental impact of *BRAF* V600E on mCRC was first reported in the early 2000s [1, 2], poor survival outcomes of resectable *BRAF* V600E mutant CRM has not been resolved, much like two-decades long arduous journey of Odysseus depicted in the Odyssey. We believe that the NEXUS trial is a crucial step in developing an effective and safe treatment for resectable *BRAF* V600E mutant CRM, which will eventually help end the odyssey.

## Data Availability

Data sharing is not applicable to this paper.

## Abbreviations

CRM: Colorectal oligometastases
CR: Complete response
ctDNA: Circulating tumor DNA
DFS: Disease-free survival
PFS: Progression-free survival
OS: Overall survival
ORR: Objective response rate
HR: Hazard ratio
mCRC: Metastatic colorectal cancer
